# Using advanced statistical tools to assess the impact of a small landfill site on the aquatic environment

**DOI:** 10.1007/s10661-021-08850-4

**Published:** 2021-01-19

**Authors:** Grzegorz Przydatek

**Affiliations:** Engineering Institute, State University of Applied Sciences in Nowy Sącz, Zamenhofa 1a street, 33-300 Nowy Sacz, Poland

**Keywords:** Municipal waste, Impact, Landfill, Water, Leachate, Advanced statistical analysis

## Abstract

The aim of the study was to assess the impact of a small municipal landfill on the aquatic environment over 9 years, using advanced statistical tools. The results of the study of surface, ground- and leachate waters from 2008 to 2016 were subjected to detailed statistical analysis based on 15 physicochemical indicators. Factor analysis accounted for the requirements of the WHO, the European Union and the nation of Poland using 8 statistical analytical methods. The analysis of leachate contamination from the landfill site with the use of advanced statistical tools revealed its interaction with groundwater. The assessment was based on increased and statistically significant values and correlations of temperature, Zn and N–NO_3_ between leachate and groundwater, factors demonstrating the negative impact of the landfill. In the case of Zn, there was also a correlation between the tested waters below the landfill. The increased PAH values in the examined surface and ground waters were not a consequence of waste disposal. However, the deterioration of the chemical state of groundwater in the vicinity of the landfill could result from a certain dysfunction of the facility’s infrastructure after operating for more than 20 years.

## Introduction

In general, growing material consumption has led to a massive increase in waste generation, especially municipal solid waste (MSW), and waste management has become a major problem for governments (Rajaeifara et al. 2017). One of the elements of waste management is storage. Despite the actions taken to minimize the mass of generated waste and its rational recovery, waste storage remains the most widely used waste management solution in the world (Laner et al. 2012). In 2018, a total of 52 million Mg of waste was deposited in the European Union and 12 million Mg in Poland (Eurostat 2018; Statistics Poland 2018). Waste disposal usually occurs in landfills with different forms of environmental protection organization. The functioning of landfills as engineering facilities should help to minimize their negative impact, especially on the aquatic environment. Such a process should be fulfilled by locating, constructing and operating landfills in a manner that accounts for hydrological and geotechnical conditions (Przydatek 2019a).

One of the side effects of waste disposal is the leachate produced as a result of rainwater migration through the deposited waste, which rinses out dissolved organic and mineral substances. During migration, organic, inorganic, colloidal, pathogenic and other contaminated substances are transferred in the waste (Zin et al. 2012), which is characterised by diverse chemical composition, depending primarily on the waste composition (Singh et al. 2012). The age of the landfill, the amount of precipitation, seasonal weather variability and storage technology also influence the quality of the leachate (Kjeldsen et al. 2002; Singh et al. 2016).

The leachate from municipal landfills can be a potential source of surface water, groundwater and soil pollution (Barbieri et al. 2014; Aziz et al. 2015), as it is the longest emitting pollutant generated in landfills. If it is not adequately protected (Patil et al. 2013), the migration of leachate from landfills, in particular, poses a high risk to groundwater resources (Chen et al. 2019). Effluents can enter groundwater aquifers as a result of precipitation and be transferred into the adjacent river system through groundwater flow, possibly polluting the surrounding environment (Naveen et al. 2018). They may contain multiple mineral and organic compounds, the amount of which should be systematically controlled (Przydatek 2019b). In this context, the guidelines of Council Directive 1999/31/EC of 26 April 1999 oblige EU member states to comply with environmental laws and regulations on the monitoring of landfills, including water and leachate.

The assessment of the impact of landfills on the quality of surface water and groundwater needs to account for the selection of indicators, which should be the same for the types of water tested and the leachate. Therefore, it is beneficial to analyse the impact of landfills on the environment by using statistical tools, which make it possible to significantly increase the probability of detecting the degree and causes of the negative impact of landfilled waste on the water environment (Atta et al. 2015; Aziz et al. 2018; Koda et al. 2017; Przydatek and Kanownik 2019; Przydatek 2019a; Srivastava and Ramanathan 2008; Tałałaj 2014). The use of a wide range of statistical tools assists in classifying, modelling and interpreting large data sets, which allows for a reduction in the form of data extraction, helping to assess water quality (Gibrilla et al. 2011; Singh et al. 2016).

The aim of the study was to assess the impact of a small municipal landfill site located in the vicinity of a river in an organised form, on the water environment using advanced statistical tools.

## Materials and methods

### Study objective

**Fig. 1:**
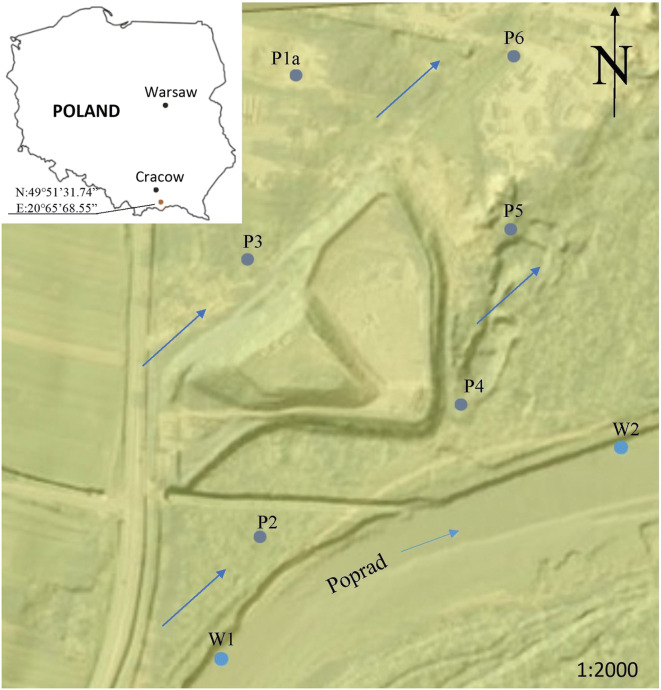
Location of reached points in the around of the municipal solid waste landfill site in XY (Southern Małopolska, Poland): points of groundwater sampling (P1a, P2, P3, P4, P5, P6) and surface water sampling (W1, W2), and direction of surface water flow is shown with bright blue arrows and groundwater of flow is shown with blue arrows

The studied 1.45-ha landfill for non-hazardous and inert waste is located in XY (49° 51′ 31.74″ N, 20° 65′ 68.55″ E) in southern Poland, several meters from the Poprad riverbed (Fig. 1). At the beginning of the 1990s, the superstructure of the existing municipal landfill was thoroughly modernized with the accompanying technical infrastructure. After the modernisation, operations began in 1999.

The area of the landfill is located within the Magura Nappe, composed of Cretaceous and Paleogene period deposits, i.e. sandstone and shale (typical flysch formations). There are Tertiary formations of deeper subsoil covered with Holocene river formations, developed in the form of pebbles, gravels, sand and gravel mix, and sands with thin interlayer loams.

**Fig. 2:**
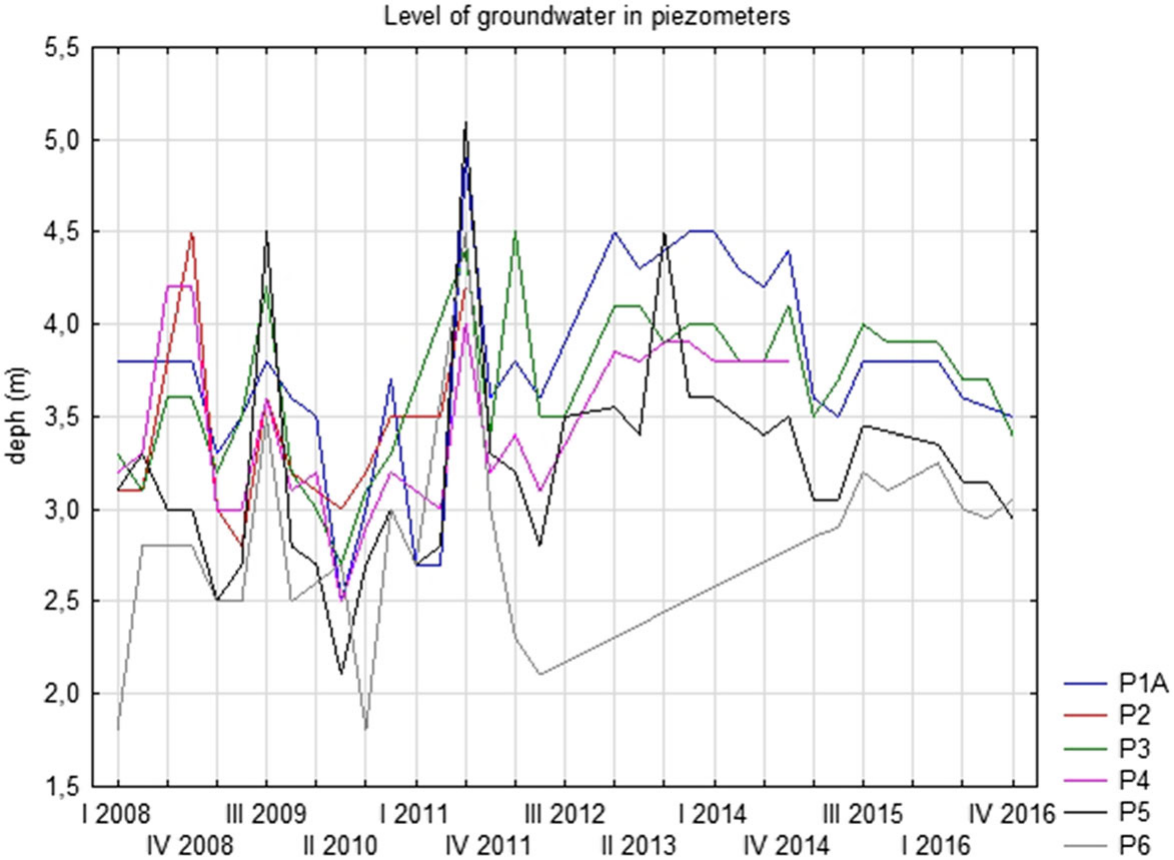
Level of groundwater in piezometers

In the vicinity of the landfill, groundwater is found in flysh and Quaternary formations. In flysch formations, water is contained in sandstone layers of bedrock, the amount of which depends on the size of the sandstone crevices that contact each other and the sandstone porosity. In Quaternary formations, the main aquifer occurs in Holocene stone and gravel formations of the Poprad River terraces. These waters are hydraulically connected with the waters of the river, the valley of which is a system that drains underground water flowing down the mountain slopes (Przydatek 2019a). Generally, the groundwater flow is directed towards the Poprad from the southwest to the northeast (Figs. 1 and 2).The landfill area consists of two sectors, the first of which remains in the rehabilitation phase, while the second (134,932 m^3^) is in operation. Within the landfill, there is a stable embankment that is 2.0–5.0 m high. The bottom of these two sectors of the landfill has been sealed with synthetic insulation in the form of a bentonite mat, 2.5-mm-thick HDPE geomembrane and geotextile to protect the soil and water environment. The landfill leachate is captured by a Ø 100-mm drain and a collective drainage system with a Ø 200-mm diameter and is collected in an 18.3-m^3^ tank prior to transport to a sewage treatment plant. This landfill has a passive degassing system consisting of five wells. In the first sector, there are two wells, and in the second sector, three wells. The degassing wells are made of corrugated perforated pipes placed into waste.

Residual waste after segregation is sent to a landfill site due to the required minimisation of the amount of deposited waste, which should be beneficial for the reduction of potential environmental hazards. The waste deposited at the landfill site is separated and thickened with a bulldozer in thin layers, 0.3–0.5-m-thick to 1.2-m high, and then systematically transferred with an insulation layer with a maximum thickness of 0.3 m (Przydatek 2019a).

### Scope of research

The results of physicochemical elements of surface water and groundwater, leachate, precipitation and amounts of leachate and waste collected from 2008 to 2016 were used to conduct statistical analyses and draw conclusions aimed at recognising the impact of a small landfill with an organised form on the quality of water in its immediate vicinity.

Samples for surface water testing were collected in the Poprad riverbed (springs in Slovakia) at two points: W1 above and W2 below the landfill. There are six piezometers within the landfill from which water samples for qualitative and quantitative studies were taken. Two piezometers P2 and P3 (reference points) are located above the landfill on the groundwater inflow. The other four are located below the landfill (P1a, P4, P5 and P6) and on the groundwater outflow (Fig. 1). It should be noted that the location of the P1a piezometric point indicates that it is out of the reach of the landfill. The sampling of groundwater from piezometers P2 and P4 in the analysed years was characterised by a certain irregularity as a result of the periodic lack of water at these points.

At piezometric points, groundwater samples were collected, and their depth measured following pumping. The leachate water was collected from the tank before it was cleaned. After samples of water and leachate were collected in the field, pH and electrical conductivity were measured using a portable multifunctional meter with glass electrodes. The meter was calibrated before each time before the research was conducted. Each result was based on the average of three measurements. The samples were collected in sterilised polyethene containers and delivered immediately to an accredited testing laboratory for analysis according to standard methods. The minimum amount of the sample taken was 500 mg/L. The samples taken were transported to the laboratory at 4 °C in the dark (APHA 2007).The concentrations of the following pollutants were determined: copper (Cu), cadmium (Cd), chromium (Cr^+6^), mercury (Hg), N–NO_3_, total organic carbon (TOC) and polycyclic aromatic hydrocarbons (PAHs). Heavy metals Cu, Cd, Cr^+6^ and Hg were determined by atomic absorption spectroscopy (AAS), which uses the phenomenon of atomic absorption of electromagnetic radiation. TOC and N–NO_3_ were determined by spectrophotometry, while HPLC with fluorescent detection after liquid-liquid extraction was used to determine PAHs. The biochemical oxygen demand (BOD5) was determined by the dilution method, with the addition of inoculum material and allylthiourea, and COD by test kits pre-made by the manufacturer HACH. Laboratory analyses were performed twice (repeated) when acceptable values were exceeded, or results outside the calibration coefficient or atypical results for a given matrix were obtained. The content of the leachate chemical contamination index shown was determined to the nearest μg/L.

The quality of surface waters was determined per the Regulation of the Minister of Environment of 21 July 2016 on the method of classification of the status of surface water bodies and the ME Regulation (2016) environmental quality standards for priority substances. Groundwater in piezometers was determined per the Regulation of the Minister of Maritime Economy and Inland Navigation of 11 October 2019 on criteria and method of assessment of the status of groundwater bodies (MMEIN Regulation 2019a). The results of the tests of leachate water from the landfill were compared with those in the Regulation of the Minister of Maritime Economy and Inland Navigation of 12 July 2019 on substances particularly harmful to the aquatic environment and the conditions to be met when discharging sewage into waters or soil and when discharging rainwater or snowmelt into waters or water facilities (MMEIN Regulation 2019b).

The verification of exceeding the maximum permissible pollutant value for leachate from the landfill and the quality class of surface and groundwater in piezometers was determined based on the arithmetic mean value for each of the tested physicochemical elements. In addition, the results of the tested waters were compared with the limit values set by the WHO (2017) for drinking water.

The quarterly sum of precipitation and the average quarterly flow of the Poprad were determined. Meteorological data were obtained from meteorological stations of the Institute of Meteorology and Water Management (49° 37′ 38″ N, 20° 41′ 21″ E) located in the vicinity of the landfill, i.e. close to the riverbed of the studied river (49° 34′ 05.65″ N, 20° 39′ 35.83″ E) and in the neighbouring village (49° 37′ 38″ N, 20° 41′ 21″ E) (Przydatek 2019b).

### Statistical analyses

For the collected results of the tested leachate and water including physical and chemical variables (reaction temperature, specific electrolytic conductivity, 5-day BOD5, COD, TOC, total nitrogen (N), N–NO_3_, PAH, Cu, Cd, Cr^+6^, Hg) and surface water flow, groundwater table depth, amount of precipitation and amount of leachate and waste, we determined the following: minimum, maximum, arithmetic mean and standard deviation (groundwater depth only). Many of the demonstrated physicochemical indicators in the study of surface water and groundwater quality in the landfill area were used by Tałałaj (2013).

For the calculation of some values of statistical parameters, the measurement result at half of a given limit of quantification was assumed when the value of water indicators in a given sample was below the limit of quantification defined by a multiple of detectability, i.e. the output signal or concentration value above which it can be stated with relative certainty that the sample differs from a blank value (Przydatek 2019a; Przydatek and Kanownik 2019).

The ANOVA test was used to estimate the significance of the differences in the concentrations of the tested surface and groundwater indices above and below the municipal landfill. As most of the variable decomposition conditions (excluding pH, electrolytic conductivity and temperature) were not met, the Kruskal-Wallis test (the non-parametric equivalent of the ANOVA test) and the median test as well as multiple (bilateral) comparisons of mean ranks, which do not require normal decomposition or homogeneous variations, were used. The Mann-Whitney *U* test was used to investigate the significance of the differences in the concentrations of the tested waters due to failure to meet the Student’s *t* test assumptions (except for pH, electrical conductivity and temperature). If the null hypothesis was rejected in the analysis of variance, the significance of the differences between the individual averages was examined using multiple post hoc comparison tests (this element exists only if the differences in the ANOVA test are statistically significant). Correlations were determined between the composition of leachate with water taken from the watercourse and piezometers located below the landfill to determine the impact of a municipal landfill on the physicochemical state of surface water and groundwater. Pearson’s correlation coefficient method was used to determine the correlation relationship for normally distributed data. When the condition of normal distribution was not met, the Spearman rank method was used, wherein the Spearman *R* correlation coefficient is the non-parametric equivalent of Pearson’s coefficient. Rank correlation shows any monotonic (or non-linear) dependence. As in the case of parametric correlation, the Spearman *R* correlation coefficient measures the strength of the relationship between the variables, but in this case, a quantitative scale with normal distribution is not applicable.

The non-parametric tests were applied due to the lack of normal distribution of most of the analysed physicochemical indicators according to the Shapiro-Wilk test results and the lack of equality of variance determined by the Mann-Kendall test. For the physicochemical elements of the examined waters, significantly different from each other, the extreme values, median and quartile range are presented in the box plots. The Mann-Kendall non-parametric statistical test was chosen for testing multiple figures to identify an upward or downward trend, which was not necessarily linear. A test diagram was also used, which is one of the simplest types of graphs, i.e. a linear graph for cases. Cluster analysis was also used to explain the detection of the structure in the data, taking into account the hierarchical clustering, which allows for the determination of the so-called *tree structure* of elements of the analysed set of objects. For all statistical analysis, the Statistica 13 programme was used (StatSoft Polska, StatSoft, Inc., USA).

## Results

### The qualitative-quantitative analysis of surface water, groundwater, leachate and deposited waste

The average quarterly amount of leachate from the landfill in the years 2008–2016 was 658.29 m^3^, and the average quarterly precipitation was 194.15 mm. According to Enekwechi and Longe (2007), the amount of leachate produced is highly dependent on the surface of the landfill, meteorological and hydrogeological factors and the tightness of the cover. In turn, the average amount of waste deposited was 658.29 Mg, at total mass of 5266.32 Mg, which showed in the analysed period was deposited less than 10 Mg waste per day (Table 1).

**Table 1:** Scope and average of meteorological, waste and leachate volumes

Indicator	Unit	Min	Max	Average
Amount of waste	Mg	0.00	1273.92	658.29
Amount of leachate	m^3^	0.00	2511	699.304
Precipitation	mm	51.1	524	194.15

Per ME Regulation (2016) and based on the analysis of quarterly results of physicochemical water quality of the river flowing in the vicinity of a landfill site, samples from two test points (W1 and W2) met most class I water quality standards. The exceptions were electrical conductivity, BOD5, COD, N and N–NO_3_ (Table 2). Based on the average value of electrical conductivity, the level of geochemical background and the level of purity, class II for electrical conductivity was exceeded by 0.07 mg/L. Similarly, measurements of other variables showed that the limit values of the second class were exceeded. Furthermore, the maximum concentration of 75.5 mg/L of N–NO_3_ at the surface water inflow exceeded the WHO limit value (2017).

**Table 2:** Scope and average values of physicochemical elements of surface water and quality class

Physicochemical elements	Unit	Examined point						Classification quality of surface water		
		W1		W2		W1	W2	Regulation ME (2016)		WHO (2017)
		Min	Max			Average		I	II	
Flow	m^3^/s	4536	74,844	4536	74,844	21,580.17	21,656.78	–	–	–
General elements										
pH		7.3	8.9	7.2	9	8.1	8.1	8.1–8.6	7.3–8.6	–
Temperature	°C	− 0.5	19.3	− 0.5	19.3	10.17	10.12	≤ 22	≤24	–
EC	mS/cm	0.23	0.52	0.23	0.54	0.37	0.37	≤ 0.27	≤0.3	–
TOC	mg/L	1.2	9.1	1.5	8.1	3.2	3.33	≤ 1.0	≤2.0	–
Inorganic elements										
Lead	mg/L	< 0.0001	< 0.05	< 0.0004	< 0.05	0.004	0.004	–	–	0.01
Cadmium	mg/L	< 0.0001	< 0.0001	< 0.0001	< 0.0001	0.0007	0.0007	–	–	0.003
Copper	mg/L	< 0.0008	< 0.02	< 0.0008	0.012	0.003	0.004	≤ 0.05		2
Zinc	mg/L	< 0.05	0.078	0.003	0.14	0.03	0.03	≤ 1		–
Chromium (VI)	mg/L	< 0.006	< 0.05	< 0.006	< 0.05	0.006	0.006	≤ 0.02		0.05
Mercury	mg/L	< 0.00005	0.001	< 0.00005	0.005	0.0002	0.0003	–	–	0.006
COD	mg/L	5	85	5	85	16.36	16.83	≤ 10	≤15	–
BOD5	mg/L	0.73	16	0.56	16	2.29	2.42	≤ 1.0	≤2.0	–
Nitrates	mg/L	0.03	75.5	0.03	18.9	5.43	5.48	≤ 0.8	≤0.9	50
Total nitrogen	mg/L	0.79	101.0	0.81	101.0	5.24	5.60	≤ 0.9	≤1.3	–
Organic elements										
PAH	μg/L	0.009	0.42	0.01	0.19	0.06	0.04	–	–	–

The analysis of the quarterly results of groundwater quality in six piezometers located in the area of the landfill site over 9 years, taking into account the limit values set out in the MMEIN Regulation (2019), showed that most of the examined physicochemical elements meet the class I standards of very good water quality. Average pH values between 7.1 and 7.3 were measured at both the inflow and outflow of water. Above the landfill, groundwater quality deteriorated significantly. It was classified as the worst (class V) due to the high average PAH concentrations, significantly exceeding the 0.0005 mg/L limit value in two piezometers (P2 and P3). All the remaining variables indicated class II (good quality). They did not meet class I quality due to exceeding the average TOC concentration by almost 5 mg/L, the average electrical conductivity by 0.2 mS/cm, and Cr^+6^ by 0.01 mg/L. Class II water quality was also found to result from more than two times the average N–NO_3_ concentration, at P3 (Table 3). Below the landfill, underground water, due to average PAH concentrations significantly exceeding the level acceptable in four piezometers at the same time, was classified as class V, the same as at the inflow. In contrast, the waters below the landfill were classified as class III, not meeting the average concentration of N–NO_3_ in P1a required for class II by 3.85 mg/L. The water level in this piezometer was stable, which was confirmed by the lowest standard deviation (SD = 0.000). The waters below the landfill site were class II, slightly exceeding the requirements of class I for temperature at P1a, P5 and P6 by 1.21, 0.65 and 0.34 °C, respectively. The average Zn concentration exceeded by 0.03 mg/L in P5. The piezometers P1a and P5 had the highest Zn values of 0.390 and 0.198 mg/L, respectively (Table 4). In comparison, the average Cd concentration below the landfill was 0.001 mg/L, and the average value of electrical conductivity at the outflow was the same as the inflow water.

**Table 3:** Scope and average values of physicochemical elements of groundwater quality class on inflow

Physicochemical elements	Unit	Piezometer						Limit value in classes (Regulation MMEIN 2019)						
		P2		P3		P2	P3	Hydrochemical						WHO (2017)
		Min	Max			Average		background	I	II	III	IV	V	
Groundwater depth	m	2.8	4.5	2.7	4.5	3.4	3.7	–	–	–	–	–	–	–
General elements														
pH		6.9	7.9	6.7	7.5	7.3	7.1	6.5–8.5	6.5–9.5			< 6.59 or	> 9.5	–
Temperature	°C	6.0	16.0	7.2	16.7	10.4	11.0	4–20	< 10	12	16	25	> 25	–
EC	mS/cm	0.6	1.1	0.6	1.0	0.9	0.8	0.2–0.7	0.7	2.5	2.5	3	> 3.0	–
TOC	mg/L	2.3	57	0.05	38.5	9.97	4.16	1–10	5	10	10	20	> 20	–
Inorganic elements														
Lead	mg/L	< 0.004	< 0.05	< 0.008	< 0.05	0.008	0.004	0.001–0.010	0.01	0.025	0.1	0.1	> 0.1	0.01
Cadmium	mg/L	< 0.0001	0.001	< 0.0001	< 0.01	0.001	0.001	0.0001–0.0005	0.001	0.003	0.005	0.01	> 0.01	0.003
Copper	mg/L	0.005	< 0.008	< 0.008	< 0.002	0.007	0.004	0.001–0.020	0.01	0.05	0.2	0.5	> 0.5	2
Zinc	mg/L	< 0.01	0.19	< 0.01	0.21	0.05	0.05	0.005–0.050	0.05	0.5	1	2	> 2	–
Chromium (VI)	mg/L	< 0.006	< 0.10	< 0.006	< 0.10	0.011	0.008	0.0001–0.010	0.01	0.05	0.05	0.1	> 0.1	0.05
Mercury	mg/L	< 0.0001	< 0.00075	< 0.00005	0.0032	0.0003	0.0003	–	0.001	0.001	0.001	0.005	> 0.005	0.006
COD	mg/L	–	–	5.00	151.00	–	24.18	–	–	–	–	–	–	–
BOD5	mg/L	–	–	0.25	57.6	–	4.58	–	–	–	–	–	–	–
Nitrates	mg/L	–	–	7.11	35.10	–	19.65	0–5	10	25	50	100	> 100	50
Total nitrogen	mg/L	–	–	2.96	7.93	–	4.81	–	–	–	–	–	–	–
Organic elements														
PAH	μg/L	< 0.02	0.520	< 0.02	0.460	0.080	0.076	0.000001–0.0001	0.0001	0.0002	0.0003	0.0005	> 0.0005	–

**Table 4:** Scope and average values of physicochemical elements of groundwater quality class on outflow

Physicochemical elements	Unit	Piezometer												Limit value in classes (Regulation MMEIN 2019)						
		P1a		P4		P5		P6		P1	P4	P5	P6	Hydrochemical						WHO (2017)
		Min	Max							Average				background	I	II	III	IV	V	
Groundwater depth	m	2.50	4.90	2.50	4.20	2.10	5.10	1.80	4.50	3.76	3.46	3.24	2.81	–	–	–	–	–	–	–
General elements																				
pH		6.7	7.7	6.6	8.1	6.6	7.9	6.7	8.4	7.1	7.1	7.2	7.3	6.5–8.5	6.5–9.5			< 6.59 or	> 9.5	–
Temperature	°C	6.3	17	4.90	15.6	6	15.4	3.90	16.20	11.21	9.94	10.65	10.34	4–20	< 10	12	16	25	> 25	–
EC	mS/cm	0.5	1.3	0.5	1.2	0.4	1.1	0.3	1.6	0.8	0.9	0.7	0.8	0.2–0.7	0.7	2.5	2.5	3	> 3.0	–
TOC	mg/L	< 0.036	14.60	< 0.5	12	0.50	16	< 1	12	2.74	3.83	3.91	3.85	1–10	5	10	10	20	> 20	–
Inorganic elements																				
Lead	mg/L	0.0002	< 0.050	0.0002	< 0.050	0.0002	< 0.05	0.0002	< 0.050	0.0043	0.0046	0.0039	0.0045	0.001–0.010	0.01	0.025	0.1	0.1	> 0.1	0.01
Cadmium	mg/L	< 0.0001	< 0.010	< 0.0001	< 0.010	< 0.0001	< 0.010	< 0.0001	< 0.010	0.001	0.001	0.001	0.001	0.0001–0.0005	0.001	0.003	0.005	0.01	> 0.01	0.003
Copper	mg/L	< 0.002	0.019	< 0.002	< 0.02	< 0.002	0.013	< 0.002	< 0.02	0.004	0.004	0.004	0.004	0.001–0.020	0.01	0.05	0.2	0.5	> 0.5	2
Zinc	mg/L	< 0.010	0.390	< 0.020	0.270	< 0.020	0.198	0.002	0.034	0.050	0.048	0.053	0.047	0.005–0.050	0.05	0.5	1	2	> 2	
Chromium (VI)	mg/L	< 0.006	< 0.10	< 0.006	< 0.10	< 0.006	< 0.10	< 0.006	< 0.10	0.009	0.011	0.009	0.010	0.0001–0.010	0.01	0.05	0.05	0.1	>0.1	0.05
Mercury	mg/L	< 0.00005	< 0.0001	< 0.00005	0.00110	< 0.00005	0.0011	< 0.00005	< 0.0015	0.00018	0.00023	0.00019	0.00019	–	0.001	0.001	0.001	0.005	> 0.005	0.006
COD	mg/L	< 10	< 10	< 10	132	< 10	263	< 10	16	5	22.58	21.52	9.50	–	–	–	–	–	–	–
BOD5	mg/L	< 0.50	1.6	< 0.5	7.1	< 0.5	8.2	< 0.5	0.9	0.43	1.79	1.49	0.56	–	–	–	–	–	–	–
Nitrates	mg/L	15.20	39.70	0.01	45.40	0.01	21.40	< 4.5	16.3	25.85	17.64	5.39	4.58	0–5	10	25	50	100	> 100	50
Total nitrogen	mg/L	4.07	9.17	1.72	31.80	< 0.50	11.60	< 0.5	4.26	6.10	6.05	2.30	1.11	–	–	–	–	–	–	–
Organic elements															–	–	–	–	–	–
PAH	μg/L	< 0.02	0.520	< 0.02	1.77	0.008	0.749	< 0.02	< 1.00	0.035	0.114	0.090	0.081	0.000001–0.0001	0.0001	0.0002	0.0003	0.0005	> 0.0005	–

In leachate from the municipal landfill, most of the average concentrations of the examined indicators were lower than the maximum normative values (MMEIN Regulation, 2019). One such indicator is the pH, the average value of which was near neutral. However, leachate from the landfill site did not meet the requirements for the introduction of wastewater into the water or the ground due to the high concentration of TOC, which ranged from 16 to 560 mg/L with an average of 61.40 mg/L, twice as high as the limit value. Landfill leachate was also characterised by a high COD of 4.50 to 300 mg/L with an average of 135.92 mg/L, which exceeded the permitted level by 10.92 mg/L. The average BOD5 concentration was lower than permissible by 13.85 mg/L. The COD/BOD ratio provided a biodegradability index of 0.08. Average concentrations of nutrients, including total N (87.19 mg/L) and N–NO_3_ (134.50 mg/L), showed that the limit values were exceeded by 3 and 4 times, respectively. The PAH concentration in leachate was low (average of 0.109 μg/L). The highest concentrations of heavy metals were low, between 0.003 and 0.68 mg/L, the lowest of which was for Hg and the highest for Zn (Table 5).

**Table 5:** Statistical parameters describing values of pollution indicators in the leachate from municipal solid waste landfill site and admissible values

Pollution indicators	Unit	Min	Max	Average	The highest admissible values in accordance with Regulation MMEIN (2019)
pH		7	9	7	6.5–12.5
Temperature	°C	5.80	19.90	12	35
EC	mS/cm	1.34	8.20	3.91	–
TOC	mg/L	16	560	61.40	30
Lead	mg/L	0.0003	< 0.05	0.02	0.5
Cadmium	mg/L	< 0.0005	0.005	0.002	0.4
Copper	mg/L	< 0.005	0.03	0.01	0.5
Zinc	mg/L	< 0.025	0.68	0.06	2
Chromium (VI)	mg/L	< 0.006	0.05	0.01	0.1
Mercury	mg/L	0.0001	0.003	0.0004	0.06
COD	mg/L	4.50	300	135.92	125
BOD5	mg/L	1.60	34	11.15	25
Nitrates	mg/L	0.92	629	134.50	30
Total nitrogen	mg/L	3.20	222	87.19	30
PAH	μg/L	0.004	2.420	0.109	–

### Analysis of research results using advanced statistical tools

Statistical comparative analysis of 12 physicochemical variables of surface waters including TOC, Pb, Cd, Cu, Zn, Cr^+6^, Hg, BOD5, COD, N, N–NO_3_ and PAHs with the Whitney-Mann *U* test showed no significant differences (*p* > 0.05).

**Fig. 3:**
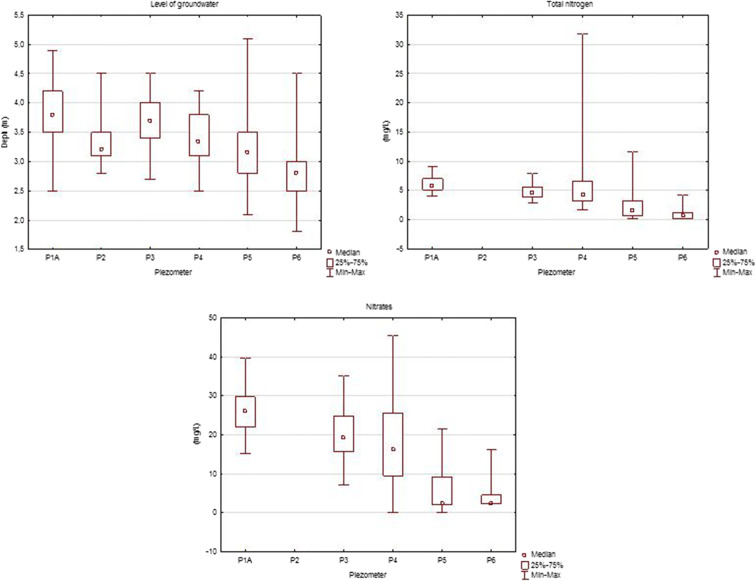
Differences between piezometers

In contrast, a comparative analysis of groundwater physicochemical elements by the Kruskal-Wallis non-parametric test and the median and multiple post hoc repetition tests showed that only seven indicators, i.e. groundwater depth, electrical conductivity, BOD5, TOC, N, N–NO_3_ and PAHs, differed significantly between piezometers at a significance level of *α* = 0.05 (Table 6). Differences in the values of physicochemical elements in groundwater were found between piezometer P3 located at the inflow and piezometers P4, P5 and P6 at the outflow of groundwater. In piezometers P5 and P6, a statistically significant lower depth of groundwater was found at the outflow of water than in piezometer P3 at the inflow. Moreover, the nitrate value in the P4 piezometer was significantly higher than in P3. The concentration of total nitrogen below the landfill in piezometer P3 was significantly lower than in groundwater at the inflow in piezometers P4 and P5 (Fig. 3). These physicochemical variables, in addition to the depth of water, cause deterioration of groundwater quality with significantly higher values at point P4. To assess the impact of the landfill on the physicochemical state of surface and groundwater, Spearman’s *R* test was used to analyse the correlation between water in the mountain river (W2) and piezometers below the landfill (P4, P5, P6) and leachate from the landfill, taking into account the variables mentioned above (*p* < 0.05).

**Table 6:** Comparison of physicochemical elements values between piezometers using non-parametric Kruskal-Wallis test

Physicochemical elements	Piezometres	P1a	P2	P3	P4	P5	P6
Groundwater depth (m)	P1a		0.3881	1.0000	0.9866	*0.0007*	*0.0000*^*a*^
	P2	0.3881		0.9471	1.0000	1.0000	0.0926
	P3	1.0000	0.9471		1.0000	*0.0052*	*0.0000*
	P4	0.9866	1.0000	1.0000		0.7584	*0.0007*
	P5	*0.0007*	1.0000	*0.0052*	0.7584		0.2443
	P6	*0.0000*	0.0926	*0.0000*	*0.0007*	0.2443	
EC (mS/cm)	P1a		0.610480	1.000000	0.224188	*0.010076*	1.000000
	P2	0.610480		0.242471	1.000000	*0.000045*	0.341130
	P3	1.000000	0.242471		0.061949	0.056110	1.000000
	P4	0.224188	1.000000	0.061949		*0.000000*	0.116202
	P5	*0.010076*	*0.000045*	0.056110	*0.000000*		0.088865
	P6	1.000000	0.341130	1.000000	0.116202	0.088865	
BOD5 (mg/L)	P1a			1.000	*0.004*	*0.003*	1.000
	P3	1.000			0.442	0.442	1.000
	P4	*0.004*		0.442		1.000	0.383
	P5	*0.003*		0.442	1.000		0.397
	P6	1.000		1.000	0.383	0.397	
COD (mg/L)	P1a			0.0560	*0.0002*	*0.0027*	1.0000
	P3	0.0560			1.0000	1.0000	1.0000
	P4	*0.0002*		1.0000		1.0000	0.8212
	P5	*0.0027*		1.0000	1.0000		1.0000
	P6	1.0000		1.0000	0.8212	1.0000	
TOC (mg/L)	P1a		*0.001*	1.000	0.158	0.179	*0.043*
	P2	*0.001*		0.134	1.000	0.631	1.000
	P3	1.000	0.134		1.000	1.000	1.000
	P4	0.158	1.000	1.000		1.000	1.000
	P5	0.179	0.631	1.000	1.000		1.000
	P6	*0.043*	1.000	1.000	1.000	1.000	
Total nitrogen (mg/L^1^)	P1a			1.000	0.551	*0.0000*	*0.0000*
	P3	1.00000			1.000	*0.0018*	*0.0038*
	P4	0.55071		1.000		*0.0017*	*0.0053*
	P5	*0.00000*		*0.002*	*0.002*		1.0000
	P6	*0.00003*		*0.004*	*0.005*	1.0000	
Nitrates (mg/L)	P1a			1.0000	0.1496	*0.0000*	*0.0003*
	P3	1.0000			1.0000	*0.0001*	*0.0203*
	P4	0.1496		1.0000		*0.0009*	0.1018
	P5	*0.0000*		*0.0001*	*0.0009*		1.0000
	P6	*0.0003*		*0.0203*	0.1018	1.0000	
PAH (μg/L)	P1a		1.000	0.085	1.000	1.000	0.054
	P2	1.000		1.000	1.000	1.000	0.912
	P3	0.085	1.000		1.000	1.000	1.000
	P4	1.000	1.000	1.000		1.000	1.000
	P5	1.000	1.000	1.000	1.000		1.000
	P6	0.054	0.912	1.000	1.000	1.000	

^a^Italic value of statistics means. that the relationship is statistically significant at *p* < 0.05

Statistical analysis of the correlation of physicochemical indicators of surface water and leachate showed that four of the examined indicators, i.e. pH, temperature, Cr^+6^ and Hg, were statistically significantly correlated below the landfill. The correlation analysis shows that the average relationship following with the highest coefficient (*r* = 0.57) occurred between point W2 and leachate in case of temperature. In the case of Cr^+6^ and Hg, the correlation coefficient did not exceed 0.50 (Stanisz 2006) (Table 7).

**Table 7:** Correlation dependence of physicochemical elements between water in point W2 and leachate from the landfill site

Parameter	Points	Correlation coefficient *R*
Leachate		
General elements		
pH	W2	*0.41*^*a*^
Temperature (°C)	W2	*0.57*
EC (mS/cm)	W2	0.34
TOC (mg/L)	W2	0.07
Inorganic elements		
Lead (mg/L)	W2	−0.25
Cadmium (mg/L)	W2	0.21
Copper (mg/L)	W2	−0.25
Zinc (mg/L)	W2	0.29
Chromium (VI) (mg/L)	W2	*0.49*
Mercury (mg/L)	W2	*0.45*
COD (mg/L)	W2	−0.16
BOD5 (mg/L)	W2	0.07
Nitrates (mg/L)	W2	0.09
Total nitrogen (mg/L)	W2	0.02
Organic elements		
PAH (μg/L)	W2	0.001

^a^Italic value of statistics means that the relationship is statistically significant at *p* < 0.05

**Fig. 4:**
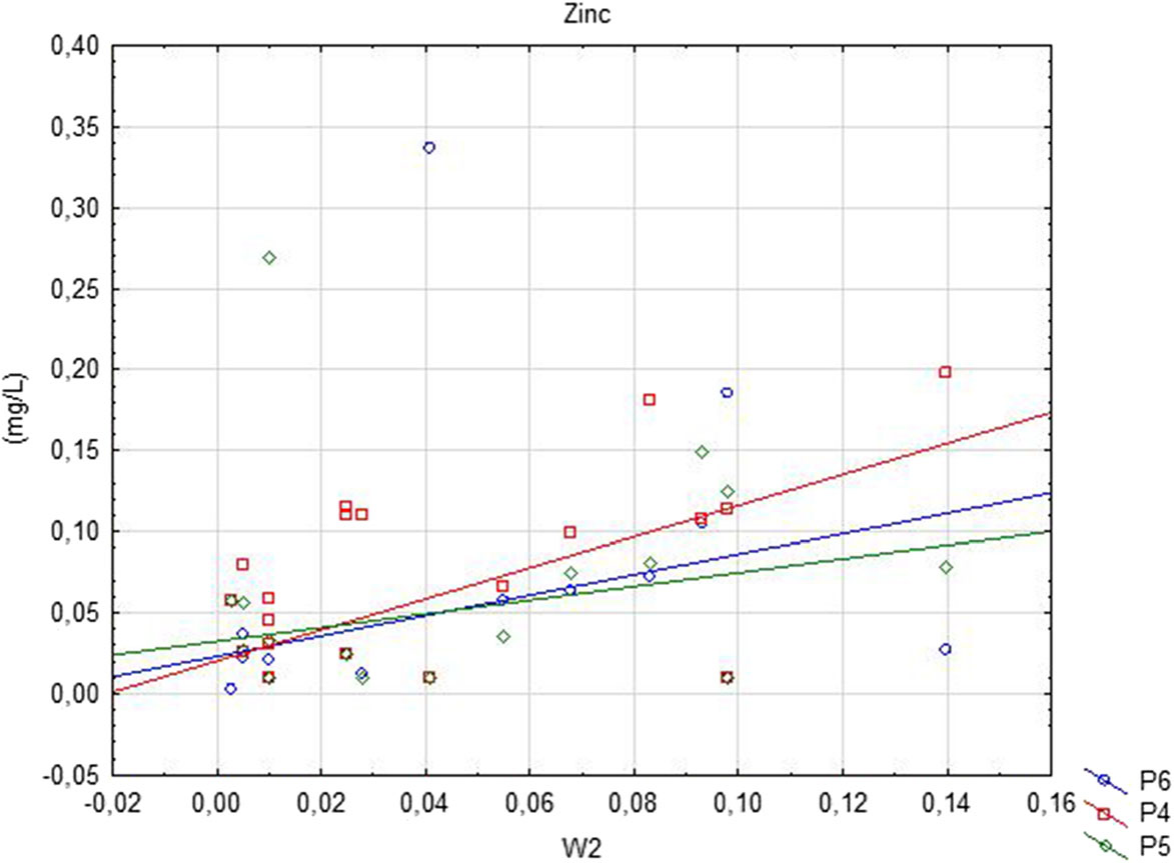
Significantly positively correlation (Zn) between groundwater and surface water below landfill site

Based on the assessment of the impact of the landfill on the physicochemical state of groundwater, a statistically negative correlation between water in the P6 piezometer and leachate was observed for BOD5. In the same piezometer, three compounds considered to be high (temperature, Cd and Zn) were significantly positively correlated between groundwater and leachate (Fig. 4). Furthermore, water in the P4 piezometer was significantly positively correlated with the leachate only in the case of two PAH and Zn designations. In contrast, underground water examined in P5 was statistically significantly correlated with leachates based on two Cd and Zn indicators at the average level. The most correlative compounds were found in point P6 below the landfill, at the most remote and at the lowest water level (Tables 2 and 8).

**Table 8:** Correlation dependence of physicochemical elements between water in piezometers P4, P5 and P6 and leachate from the landfill site

Parameter	Points	Correlation coefficient *R*
Leachate		
General elements		
pH	P4	0.19
	P5	0.20
	P6	0.40
Temperature (°C)	P4	0.44
	P5	*0.61*
	P6	*0.55*
EC (mS/cm)	P4	−0.14
	P5	0.04
	P6	− 0.13
TOC (mg/L)	P4	− 0.11
	P5	0.02
	P6	− 0.24
Inorganic elements		
Lead (mg/L)	P4	− 0.21
	P5	− 0.14
	P6	− 0.19
Cadmium (mg/L)	P4	0.37
	P5	*0.37*
	P6	*0.56*
Copper (mg/L)	P4	− 0.34
	P5	− 0.29
	P6	0.16
Zinc (mg/L)	P4	*0.52*
	P5	*0.51*
	P6	*0.62*
Chromium (VI) (mg/L)	P4	0.29
	P5	0.12
	P6	0.08
Mercury (mg/L)	P4	0.13
	P5	0.14
	P6	0.19
COD (mg/L)	P4	0.38
	P5	0.01
	P6	− 0.32
BOD5 (mg/L)	P4	− 0.10
	P5	0.21
	P6	*− 0.99*
Nitrates (mg/L)	P4	− 0.21
	P5	− 0.32
	P6	–
Total nitrogen (mg/L)	P4	− 0.01
	P5	− 0.06
	P6	− 0.22
Organic elements		
PAH (μg/L)	P4	*0.58*
	P5	− 0.01
	P6	0.07

**Fig. 5:**
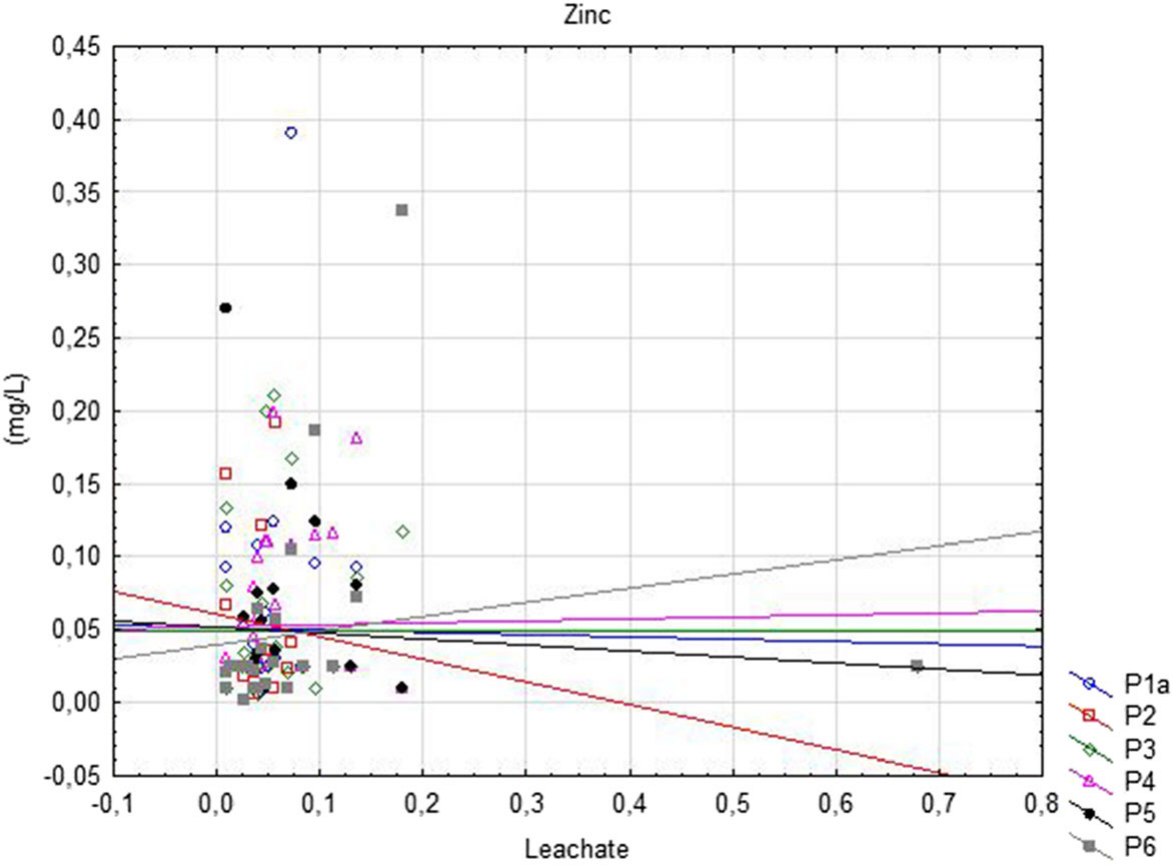
Significantly positively correlation (Zn) between groundwater and leachate below landfill site

The assessment of the relationship between the points of study of the physicochemical state of the waters below the landfill showed significant positive correlations for eight indicators. The most statistically significant variables included temperature, Pb, Cd, Cu and Hg. A strong correlation occurred between W2 and three piezometric points (P4–P6; *r* = 0.99–1.00) in Pb concentrations. There was a strong correlation between the examined points of Cd and Hg and high (P6), average (P4) and high (P5) Cu concentrations. There were also single correlation compounds taking into account N–NO_3_ as well as heavy metals Zn and Cr^+6^ (Fig. 5). The latter designations were characterised by a very high and high correlation, respectively (Table 9).

**Table 9:** Correlation dependence of physicochemical elements between water in point W2 and groundwater in piezometers P4, P5 and P6 below landfill site

Parameter	Points	Correlation coefficient *R*
W2		
General elements		
pH	P4	0.06
	P5	− 0.02
	P6	0.12
Temperature (°C)	P4	*0.60*
	P5	*0.70*
	P6	*0.87*
EC (mS/cm)	P4	− 0.04
	P5	0.14
	P6	0.06
TOC (mg/L)	P4	0.13
	P5	0.26
	P6	0.31
Inorganic elements		
Lead (mg/L)	P4	*0.99*
	P5	*1.00*
	P6	*1.00*
Cadmium (mg/L)	P4	*0.74*
	P5	*0.76*
	P6	*0.73*
Copper (mg/L)	P4	*0.44*
	P5	*0.51*
	P6	*0.91*
Zinc (mg/L)	P4	0.27
	P5	*0.67*
	P6	0.32
Chromium (VI) (mg/L)	P4	0.34
	P5	*0.36*
	P6	0.35
Mercury (mg/L)	P4	*0.54*
	P5	*0.63*
	P6	*0.62*
COD (mg/L)	P4	0.10
	P5	0.01
	P6	0.06
BOD5 (mg/L)	P4	− 0.07
	P5	0.09
	P6	− 0.65
Nitrates (mg/L)	P4	*0.71*
	P5	− 0.10
	P6	− 0.47
Total nitrogen (mg/L)	P4	0.10
	P5	− 0.08
	P6	− 0.27
Organic elements		
PAH (μg/L)	P4	0.38
	P5	0.30
	P6	− 0.17

The statistical analysis of surface water, groundwater and leachate quality testing showed mostly a statistically significant decreasing trend (*p* = 0.00–0.04) (Table 10). In surface water (W1, W2), the detected upward trend included pH significantly (*p* = 0.03–0.04). In addition, Cd (W1), COD (W2), Cu, Hg, PAH and N (W1, W2) were characterised by a significant decreasing trend (*p* = 0.000–0.012).

**Table 10:** Time trends of examined parameters quality of water and leachate

Variable	Point	Trend	Probability (*p*)
Surface water			
Cadmium	W1	↓	*0.001*
COD	W2	↓	*0.012*
Copper	W1	↓	*0.001*
Copper	W2	↓	*0.009*
Mercury	W1	↓	*0.000*
Mercury	W2	↓	*0.000*
PAH	W1	↓	*0.010*
pH	W1	↑	*0.04*
pH	W2	↑	*0.03*
Total nitrogen	W1	↓	*0.005*
Total nitrogen	W2	↓	*0.005*
Groundwater			
Groundwater depth	P6	↑	*0.022*
pH	P4	↓	*0.017*
pH	P6	↓	*0.008*
EC	P4	↑	*0.015*
Total nitrogen	P4	↓	*0.021*
COD	P3	↑	*0.014*
COD	P5	↓	*0.002*
PAH	P1A	↓	*0.035*
TOC	P1A	↓	*0.001*
TOC	P5	↓	*0.015*
TOC	P6	↓	*0.001*
Copper	P1A	↓	*0.000*
Copper	P3	↓	*0.000*
Copper	P4	↓	*0.000*
Copper	P5	↓	*0.000*
Copper	P6	↓	*0.002*
Cadmium	P1A	↓	*0.001*
Cadmium	P3	↓	*0.005*
Cadmium	P4	↓	*0.034*
Cadmium	P5	↓	*0.016*
Cadmium	P6	↓	*0.009*
Lead	P2	↓	*0.031*
Mercury	P1A	↓	*0.0000*
Mercury	P3	↓	*0.00001*
Mercury	P4	↓	*0.00005*
Mercury	P5	↓	*0.00001*
Mercury	P6	↓	*0.00004*
Nitrate	P5	↑	*0.001*
Leachate			
Total nitrogen	**–**	↓	*0.02*
Nitrates	**–**	↓	*0.001*
COD	**–**	↓	*0.04*
PAH	**–**	↓	*0.01*
Cadmium	**–**	↓	*0.000*

Italic value of statistics means that the relationship is statistically significant at *p* = 0.05

The analysis of the results of the groundwater quality survey showed a significant upward trend of variables including depth of groundwater (P6), electrical conductivity (P4), COD (P3) and N–NO_3_ (P5) at *p* = 0.001–0.022. The pH (P4, P6), N (P4), COD (P5), PAH (P1a), TOC (P1a, P5, P6), Cu and Cd (P1a–P6), Pb (P2) and Hg (P1a–P5) followed a significant decreasing trend (*p* = 0.001–0.022).

Analysis of leachate contamination confirmed only a significant decreasing trend in N, N–NO_3_, COD, PAH and Cd (*p* = 0.000–0.04).

**Fig. 6:**
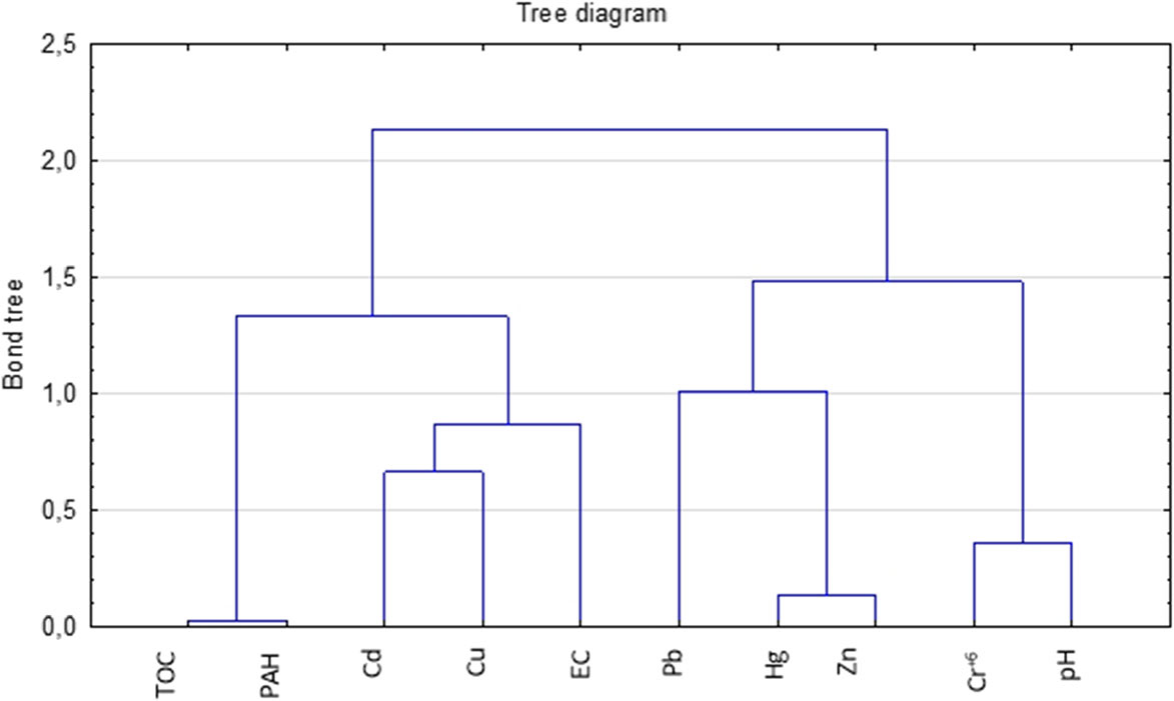
Dendrogram containing a grouping of leachate physicochemical indicators

Figure 6 shows a dendrogram containing a grouping of leachate physicochemical indicators, based on the assumption that the optimal number of clusters is two, divided into five subgroups: (1) TOC and PAHs, (2) heavy metals (Cd, Cu), (3) electrical conductivity, (4) heavy metals (Pb, Hg, Zn, Cr^+6^) and (5) pH.

## Discussion

One of the important factors affecting the composition of leachate is both the volume and composition of the deposited waste. The average amount deposited at the examined small landfill over 9 years was 658.29 Mg. At a small landfill site, Przydatek and Kanownik (2019) showed almost half the amount of waste deposited.

The deterioration of surface water quality in the vicinity of the landfill site, both above and below, was caused by values of electrical conductivity, Cr^+6^, BOD5, COD, N and N–NO_3_. In particular, the increased electrical conductivity at two points confirmed the pollution of these waters at the non-class level. Grygorczuk-Petersons and Wiater (2016) also showed a deterioration in the quality of surface water due to increased electrical conductivity. However, no statistically significant differences were found between the test points located in the riverbed below and above the landfill. Furthermore, the only upward trend detected in these waters was pH. According to Maqbool et al. (2011), the contamination of surface water is more serious than that of groundwater, because untreated leachate from the landfill can come into direct contact with streams, causing serious pollution.

The deterioration of groundwater quality in the area of the landfill site was mainly caused by increased PAH concentration significantly exceeding the acceptable level of water quality, causing it to be classified as the worst out-of-class quality, as well as by water inflow. The concentration of this organic compound class in the leachate was low, below 0.12 μg/L. Malakahmad et al. (2016) showed that PAHs originate mainly from anthropogenic processes, in particular from the incomplete combustion of organic fuels, and are widely distributed in the environment. Generally, this indicates that pollutants transported in groundwater, with a much slower flow than the surface water, can enter the latter through the inflow. Förstner and Wittmann (1979) showed that in general, groundwater advection to surface water is low; however, the concentration of pollution increases when the surface water percolates via sedimentation. The occurrence of one of these processes is indicated by increased concentration of PAHs (Przydatek and Kanownik 2019), which at high concentrations both at inflow and outflow confirms the existence of an anthropogenic source. The presence of N–NO_3_ in groundwater is also considered to be a consequence of the anthropogenic inputs (Cossu et al. 2018). Regarding the examined landfill, this confirms the deterioration of the groundwater quality at outflow due to significantly higher PAH concentrations (the lowest average exceeding 3 mg/L) and an upward trend. Ahmed and Sulaiman (2001) and Przydatek (2019a) showed a low concentration of PAHs with a significant increase in the outflow from the landfill area. Notably, the deterioration of groundwater quality was similarly affected by an increase in temperature, the highest average of which was 11.21 °C. Galarpe and Parilla (2012) showed a much higher groundwater temperature in the area of the landfill, which exceeded 25 °C. The deterioration of water quality in the area of the landfill was caused by Zn concentration in addition to the increased temperature. The most statistically significant relationships were between the temperature of groundwater and Zn, as the average concentration of Zn exceeded acceptable limits by 0.03 mg/L with the highest 0.390 mg/L, as well as the occurrence of significant correlations with leachate and surface waters below the landfill.

Among the heavy metals studied, the concentration of Zn was the highest in groundwater. Similarly, Foufou et al. (2017) observed excess Zn in the examined groundwater. Concentrations of Cr^+6^, Cd, Cu, Pb and Hg remained at a low level, similar to the neutral reaction. Kapelewska et al. (2016) observed a neutral water reaction in the area of the landfill. However, it should be noted that in the case of Cd at an average concentration of 0.001 mg/L below the landfill, there was a high correlation in the relationship between groundwater and leachate and two types of water below the landfill. Higher Cd concentrations (0.07 mg/L) in groundwater in the area of the landfill were also observed by Idrees et al. (2018). Iwuoha and Akinseye (2019) attributed significant toxicity to this element. The strongest correlations were found in the study of groundwater quality at the lowest piezometric point of the outflow, with a noticeable downward trend in the water table, which may be related to the direction of groundwater flow (Boateng et al. 2019). Other researchers found that the degree of contamination of aquifers depends on the speed of transport of the pollutants and the flow conditions at the point where they penetrate the soil structure (Vasanthi et al. 2008; Szymkiewicz et al. 2018). Han et al. (2016) showed that the negative impact of landfills could reach up to 1000 m.

The tested composition of leachate from the old landfill did not meet the requirements for the introduction of wastewater into the water or soil due to the high average concentration of TOC of 61.40 mg/L. TOC and PAH were considered to be similar relationships because they remained in the same focus. Liu et al. (2013) showed a strong correlation between these designations in surface water. The average COD concentration of 135.92 mg/L was high in the leachate.

Accordingly, Cheibub et al. (2014) considered meeting an acceptable COD to be generally possible with the combined processes of coagulation/flocculation and the Fenton process of leachate treatment from landfills. The concentration of total N was 87.19 mg/L and N–NO_3_ was 134.50 mg/L, also high values. The main component of nitrogen in leachate is usually the decomposition of complex nitrogen compounds in solid waste (Al-Yaqout and Hamoda 2020). The leachate’s biodegradability changes over time, which can be observed from the BOD5/COD ratio, for which a result below 0.1 confirms that the sample originated from an old and mature landfill (Amor et al. 2015; Kamaruddin et al. 2015). Its location near the riverbed indicates that the landfill is mature (Noerfitriyani et al. 2018). In comparison, a higher value of the biodegradability index was shown by Atta et al. (2015) in a landfill in a tropical climate. According to Kapelewska et al. (2019) and De et al. (2016), the landfills’ age significantly affects the leachate composition; hence, the heavy metals in the leachate were at a low level with the highest zinc concentration at 0.68 mg/L. In general, the concentration of metals in the analysed leachates was characterised by a downward trend.

Some researchers (e.g. De Schamphelaere et al. 2005; Heijerick et al. 2009) have noted that TOC influences the toxicity of Zn in the aquatic environment. The average Zn concentration was at a very low level, not exceeding mg/L. Boateng et al. (2019) indicated that the mean Zn concentration in the leachate at landfill in Ghana was above the acceptable level (6.092 mg/L). Similarly, Abiriga et al. (2020) showed an increased concentration of this microelement (max 5.739 mg/L) in groundwater near a landfill in Norway. The study of leachate from a landfill site used since the 1990s using statistical tools confirmed the interaction between leachate and underground and surface waters, as demonstrated in several studies (Przydatek 2019a; Przydatek and Kanownik 2019; Vahabian et al. 2019). According to Rana et al. (2018), an applied multidimensional statistical analysis is used in environmental monitoring or dataset modelling to reduce dimensionality and deviation, which is helpful in data evaluation.

The negative impact of the landfill on groundwater, according to Han et al. (2016), is considered to be most intense in the area of landfills when they are less than 20 years old. In turn, Tenodi et al. (2020) showed a negative impact on groundwater, even in a new landfill. The reason for the demonstrated negative impact after more than 20 years of landfill use could be the poor efficiency of the existing leachate treatment system, as suggested by Rowe (2005), or the leachate collection system, according to Liu et al. (2018). Another reason could be leakage of leachate through the geomembrane as a result of manufacturing and construction defects, as well as vapour diffusion through the padding (Pantini et al. 2014). Another reason for water pollution below of the landfill given by Thomsen et al. (2012) was surface runoff. However, the latter factor is unlikely to be the case because the analysed landfill has a stable embankment as indicated by the lack of leachate interaction with surface waters.

## Conclusions

Based on the 9-year analysis of the quality of the water environment in the area of a small landfill, the following conclusions may be drawn:

- The quality of water in the area of the landfill below its location resulted in changes in the classification of its quality, confirming the negative impact of a small and organized municipal landfill on groundwater quality.
- The deterioration of water quality, both above and below the landfill, was influenced by the excessive concentration of PAH, causing it to be classified as the worst non-class quality.
- The use of 8 advanced statistical methods has thoroughly analysed the occurrence of interactions between leachate and groundwater and between groundwater and surface water in the closest vicinity of the landfill.
- A significant factor in the deterioration of groundwater quality was an increase in the temperature of the tested groundwater and a positive correlation between the leachate and the groundwater and between the surface and underground waters below the landfill for toxic Zn.
- The identification of the negative impact of the landfill was also influenced by the highest number of correlations in groundwater below the landfill with regard to temperature, Cd and a significant N–NO_3_ difference.
- Most correlations were found at the lowest and outermost piezometric point at the groundwater outflow.
- The demonstrated adverse impact of the landfilled waste, despite its organized form, may have been a consequence of physical defects of the sealing screen on the ground of the landfill or limited efficiency of the leachate collection system.
